# Associations between Depressive Symptoms, Work Environment, and Lifestyle in <40-year-old Male Orthopedic Physicians in Japan

**DOI:** 10.31662/jmaj.2021-0193

**Published:** 2022-09-30

**Authors:** Akihiro Tsuchiya, Koji Wada, Natsumi Tanaka, Kazue Hayakawa, Yoji Mikami, Atsushi Okawa, Emiko Horii, Junji Ito

**Affiliations:** 1 Gender Equality and Work System Reform Committee, The Japanese Orthopaedic Association, Tokyo, Japan; 2 Department of Social Medical Sciences, Graduate School of Medicine, International University of Health and Welfare, Tokyo, Japan; 3 Department of Orthopaedic Surgery, Nagasaki Rosai Hospital, Nagasaki, Japan; 4 Department of Orthopaedic Surgery, Fujita Health University, Aichi, Japan; 5 Department of Orthopaedic Surgery, Yokohama Rosai Hospital, Kanagawa, Japan; 6 Department of Orthopaedic Surgery, Tokyo Medical and Dental University, Tokyo, Japan; 7 Department of Orthopaedic Surgery, Kansai Medical University, Osaka, Japan; 8 Department of Orthopaedic Surgery, Aomori Prefectural Central Hospital, Aomori, Japan

**Keywords:** depression, lifestyle, physicians, orthopedic surgeons, Japan

## Abstract

**Introduction:**

Efforts are being made to reduce doctors’ working hours and implement reforms in the way doctors work. This study aims to determine the associations between depressive symptoms and work environment/lifestyle among <40-year-old male orthopedic physicians in Japan.

**Methods:**

Participants were 1,343 male orthopedic physicians selected from a survey (*N* = 25,139) of all regular members conducted in 2019 by the Japanese Orthopaedic Association. Participants completed the Quick Inventory of Depressive Symptomatology and provided information about total working hours, number of on-call/night duties, number of patient complaints received, smoking habits, exercise habits, and sleep time.

**Results:**

Of the participants, 6.6% had depressive symptoms. Factors associated with depressive symptoms were total working hours of ≥80 h per week (80-99 h: adjusted odds ratio [AOR] = 2.06; 95% confidence interval [CI], 1.02-4.18; ≥100 h: AOR = 3.89; 95% CI, 1.92-7.88), one or more unreasonable patient demands/complaints in the previous 6 months (AOR = 1.61; 95% CI, 1.00-2.60), current smoking (AOR = 2.98), no sweat-inducing exercise sessions of ≥30 min per week in the previous month (AOR = 2.50), and an average of <6 h of sleep per night in the previous month (AOR = 2.15).

**Conclusions:**

Work factors at the main medical institution (i.e., total working hours of ≥80 h per week) were associated with depressive symptoms. In addition, associations between depressive symptoms and unhealthy living conditions, such as smoking habits, lack of exercise, and <6 h of sleep per night, were observed.

## Introduction

Doctors’ work environment, including working hours, affects their mental health and depressive symptoms. Clinicians have been reported to have a particularly high rate of depression. A survey conducted in 2015 by the Japan Medical Association reported that 6.5%, 0.9%, and 3.6% of physicians had moderate or high depressive symptoms, severe depression, and were at risk for suicide, respectively ^(1)^. Moreover, reports of doctors committing suicide have also been noted ^(2)^. Thus, addressing multiple factors related to working conditions to improve doctors’ work environment is necessary ^(3)^. Furthermore, addressing problems related to doctors’ mental health is also important to ensure the quality of medical care, and various proposals (e.g., standards for total working hours) have been made by the Working Style Review Committee of Doctors to reform working conditions for doctors in Japan.

Few surveys on doctors’ working conditions and mental health in the orthopedics field are available ^(4)^. Some surveys exist on the working styles of orthopedic surgeons overseas ^(5)^. Even in Japan, some surveys exist on working styles in emergency departments including other departments ^(6)^ but no survey was noted involving all orthopedic physicians in Japan. The only survey targeting orthopedic surgeons in Japan was the “Questionnaire Survey on the Current Situation of Female Doctors in the Orthopaedic Field” conducted in 2017 by the Gender Equality Committee of the Japanese Orthopaedic Association, which targeted female orthopedic surgeons ^(7)^. Results suggested that female orthopedic surgeons were stressed due to the risk of occupational exposures at life stages such as pregnancy and childbirth. The Japanese Orthopaedic Association recently conducted a questionnaire on the employment status of all members for the first time ^(8)^. The results showed that young doctors had a heavy burden. The young generation is responsible for the future of orthopedics and is likely to work long hours because they need to perform self-study in addition to their usual workload, leading to high levels of chronic stress ^(9)^. Moreover, young doctors experience chronic stress in the early stages of their careers as medical professionals and have higher turnover intentions ^(9)^. Thus, investigating and improving the work environment of the younger generation of doctors (i.e., those in their 20s and 30s) is necessary to improve the ways that physicians work.

Young orthopedic physicians are considered prone to depression because of uncontrolled work and lack of training programs ^(10)^. In addition, if a small number of female orthopedic physicians (~6% of the total) will have maternity leave, the burden will likely be placed on young male physicians. However, the cause of depression among young orthopedic physicians in Japan has not been investigated. This study aims to determine the associations between depressive symptoms, work environment, and lifestyle factors among <40-year-old male orthopedic physicians in Japan.

## Materials and Methods

The 25,139 participants who received the Japanese Orthopaedic Association questionnaire were all regular association members as of November 1, 2019, for whom the mailing addresses for the Japanese Orthopaedic Association’s journals were in Japan. A questionnaire and a reply envelope were sent to each member’s registered address. The responses were submitted either by mail or online (a link on the Japanese Orthopaedic Association website and a QR code for online responses was attached to the questionnaire). The collection period was ~3 months (from the end of November 2019 to the end of January 2020). The survey was conducted anonymously. This study has been approved by the institutional review board of the authors’ affiliated institutions.

The questionnaire was sent to 25,139 association members and 10,052 responded (response rate, 40.0%); 6,647 (66.1%) and 3,405 (33.9%) responses were received via mail and online, respectively. Participants included in this study were <40-year-old men and employed full-time (working ≥4 days per week) in medical institutions. Medical institutions included hospitals (university and nonuniversity hospitals) but excluded clinics with or without beds. After excluding ineligible participants and missing observations, 1,343 participants were finally in this study. The questionnaire covered total working hours per week at the participants’ main medical institution, number of times on night duty in the previous month at the main medical institution, number of times on call in the previous month at the main medical institution, number of unreasonable patient demands or complaints in the previous 6 months, current smoking status, number of sweat-inducing exercise sessions of ≥30 min per week in the previous month, average hours of sleep per day in the previous week, frequency of drinking alcohol per week in the previous month, years of orthopedic experience, number of orthopedic beds, number of outpatient visits per week, and average daily number of patients per week. Unanswered items were excluded as appropriate. Questions from the Quick Inventory of Depressive Symptomatology (QIDS) were included as an outcome ^(11)^. The QIDS was used to evaluate depressive symptoms. The total score is classified into five groups: normal (0-5 points), mild (6-10 points), moderate (11-15 points), severe (16-20 points), and extremely severe depression (21-27 points). Based on a previous study ^(12)^, physicians in depressive state were identified by a QIDS score ≥11.

### Statistical analysis

Simple tabulation (single analysis) was performed for each questionnaire item. Cross-tabulation of attributes and depressive symptoms as an outcome (cutoff value of 11) was next performed. Logistic regression analysis was conducted to explore the work and lifestyle factors associated with the depressive state. Crude and adjusted odds ratios were estimated with 95% confidence intervals. Total working hours, number of night duties, number of on-call duties, number of unreasonable patient demands, smoking status, exercise, sleep, experience, number of orthopedic beds, number of outpatient visits, number of patients per week, and drinking habits were used as covariates in the adjusted logistic regression analysis. All analyses were conducted using EZR for Windows, version 1.37.

### Ethical consideration

Ethical considerations related to the survey were reviewed and approved by the Ethics Review Committee established by the Japanese Orthopaedic Association and the International University of Health and Welfare Ethics Committee (approval number 19-Im-012), and consent was obtained from all the respondents for utilizing the collected data for study purposes.

## Results

Table 1 shows participants’ characteristics. The examination of total working hours showed that the largest group worked 60-69 h per week at their main medical institution (26.1%), 14% worked 80-99 h, and 10.6% worked ≥100 h. In addition, 78% and 15% were on night duty one to four and five or more times, respectively, in the previous month at their main medical institution. The largest group were on call one to four times in the previous month at their main medical institution (37.4%), followed by those who were on call ≥10 times (14.8%). More than half of the participants (52.8%) had not experienced unreasonable patient demands or complaints in the previous 6 months. Most participants (87.1%) did not smoke. Finally, 6.6% of the participants had a QIDS score ≥11 points.

**Table 1.** Characteristics of Study Participants (*n* = 1,343).

Characteristic variables	*n*	(%)
Age (years)		
25-29	202	(15.0)
30-39	1,141	(85.0)
Total working hours per week at the main medical institution where one works		
<50	135	(10.1)
50-59	285	(21.2)
60-69	351	(26.1)
70-79	241	(17.9)
80-99	188	(14.0)
≥100	143	(10.6)
Number of times on night duty in the previous month at the main medical institution where one works		
None	94	(7.0)
1-4 times	1,047	(78.0)
5 times or more	202	(15.0)
Number of times on call in the previous month at the main medical institution where one works		
None	279	(20.8)
1-4 times	502	(37.4)
5-9 times	363	(27.0)
≥10	199	(14.8)
Number of unreasonable patients demands and complaints in the previous 6 months		
None	709	(52.8)
More than once	634	(47.2)
Current smoking status			
Smoke	173	(12.9)
Do not smoke	1,170	(87.1)
Number of sweating-inducing exercise sessions of ≥30 min per week in the previous month		
None	690	(51.3)
1-2 times	546	(40.7)
3-4 times	87	(6.5)
Almost every day	20	(1.5)
Average hours of sleep per day other than the night duty in the previous month		
<6	505	(37.6)
≥6	838	(62.4)
The score on the Quick Inventory of Depressive Symptomatology (QIDS)		
0-5	1,014	(75.5)
6-10	239	(17.8)
11-15	77	(5.7)
16-20	10	(0.7)
21-27	3	(0.3)

Table 2 shows the percentage of participants with a QIDS score ≥11 or <11 points for each variable. For total working hours per week at the main medical institution, 22.2% and 28.9% of those who presented with a QIDS score ≥11 points worked 80-99 and ≥100 h per week, respectively. Among those that scored ≥11 points, 24.4% were on night duty five or more times in the previous month, and 25.6% were on call 10 or more times in the previous month. In addition, 28.9% of those who scored ≥11 points smoked and 58.9% of those who scored ≥11 points slept <6 h per night.

**Table 2.** Associations between Depressive State (Quick Inventory of Depressive Symptomatology score ≥11) and Other Variables among Male Orthopedic Physicians <40 Years Old in Japan (*n* = 1,343).

Variables	In depressive state (QIDS Score ≥11)		Not in depressive state (QIDS Score <11)	
	*n* = 90	(%)	*n* = 1,253	(%)
Total working hours per week at the main medical institution where one works				
<50	3	(3.3)	132	(10.5)
50-59	9	(10.0)	276	(22.0)
60-69	17	(18.9)	334	(26.7)
70-79	15	(16.7)	226	(18.0)
80-99	20	(22.2)	168	(13.4)
≥100	26	(28.9)	117	(9.3)
Number of times on night duty in the previous month at the main medical institution where one works
None	5	(5.6)	89	(7.1)
1-4 times	63	(70.0)	984	(78.5)
≥5 times	22	(24.4)	180	(14.4)
Number of times on call in the previous month at the main medical institution where one works				
None	14	(15.6)	265	(21.1)
1-4 times	32	(35.6)	470	(37.5)
5-9 times	21	(23.3)	342	(27.3)
≥10 times	23	(25.6)	176	(14.0)
Number of unreasonable patients demands and complaints in the previous 6 months				
None	37	(41.1)	672	(53.6)
More than once	53	(58.9)	581	(46.4)
Current smoking status				
Smoke	26	(28.9)	147	(11.7)
Do not smoke	64	(71.1)	1106	(88.3)
Number of sweating-inducing exercise sessions of ≥30 min per week in the previous month				
None	66	(73.3)	624	(49.8)
1-2 times	21	(23.3)	525	(41.9)
3-4 times	2	(2.2)	85	(6.8)
Almost every day	1	(1.1)	19	(1.5)
Average hours of sleep per day other than the night duty in the previous month
<6	53	(58.9)	452	(36.1)
≥6	37	(41.1)	801	(63.9)

*QIDS* Quick Inventory of Depressive Symptomatology

Table 3 shows the results of the univariate and logistic regression analyses for each variable. The total working hours of ≥80 h per week at the main medical institution was associated with a depressive state (80-99 h: adjusted odds ratio [AOR] = 2.06; 95% confidence interval [CI], 1.02-4.18; ≥100 h: AOR = 3.89; 95% CI 1.92-7.88). Other factors associated with a depressive state were being on night duty five or more times in the previous month at the main medical institution (AOR = 1.74; 95% CI, 0.96-3.11 weakly associated), being on call 10 or more times in the previous month at the main medical institution (AOR = 1.84; 95% CI, 0.98-3.46 weakly associated), one or more unreasonable patient demands/complaints in the previous 6 months (AOR = 1.61; 95% CI 1.00-2.60), current smoking (AOR = 2.98; 95% CI, 1.74-5.09), no sweat-inducing exercise sessions of ≥30 min per week in the previous month (AOR = 2.50; 95% CI, 1.47-4.26), and an average of <6 h of sleep per night in the previous month (AOR = 2.15; 95% CI, 1.34-3.44). Frequency of drinking per week in the previous month, years of orthopedic experience, number of orthopedic beds, number of outpatient visits per week, and average daily number of patients per week were not associated with the depressive state.

**Table 3.** Univariate and Multivariable Logistic Regression Analyses for Factors Associated with Depressive State (Quick Inventory of Depressive Symptomatology score ≥11) among Male Orthopedic Physicians Aged <40 Years Old in Japan (*n* = 1,343).

Variables	In depressive state	
	Crude OR (95% CI)	Adjusted OR (95% CI)
Total working hours per week at the main medical institution where one works		
<50	0.45 (0.13-1.55)	0.49 (0.13-1.76)
50-59	0.64 (0.28-1.46)	0.75 (0.31-1.76)
60-69	ref	ref
70-79	1.30 (0.64-2.66)	1.34 (0.63-2.86)
80-99	2.34 (1.19-4.58)	2.06 (1.02-4.18)
≥100	4.37 (2.29-8.33)	3.89 (1.92-7.88)
Number of times on night duty in the previous month at the main medical institution where one works		
None	0.88 (0.34-2.24)	1.11 (0.41-3.00)
1-4 times	ref	ref
≥5 times	1.91 (1.15-3.18)	1.74 (0.96-3.11)
Number of times on call in the previous month at the main medical institution where one works		
None	0.78 (0.40-1.48)	0.80 (0.40-1.60)
1-4 times	ref	ref
5-9 times	0.90 (0.51-1.59)	0.76 (0.41-1.42)
≥10 times	1.92 (1.09-3.37)	1.84 (0.98-3.46)
Number of unreasonable patients demands and complaints in the previous 6 months		
None	ref	ref
More than once	1.67 (1.07-2.56)	1.61 (1.00-2.60)
Current smoking status		
Smoke	3.06 (1.88-4.98)	2.98 (1.74-5.09)
Do not smoke	ref	ref
Number of sweat-inducing exercise sessions of ≥30 min per week in the previous month		
None	2.64 (1.60-4.38)	2.50 (1.47-4.26)
1-2 times	ref	ref
3-4 times	0.59 (0.14-2.55)	0.65 (0.14-2.94)
Almost every day	1.32 (0.17-10.3)	1.82 (0.21-15.3)
Average hours of sleep per day other than the night duty in the previous month		
<6	2.54 (1.64-3.92)	2.15 (1.34-3.44)
≥6	ref	ref

*OR* odds ratio, *CI* confidence interval, *ref* reference Data adjusted additionally for years of orthopedic experience, number of orthopedic beds, number of outpatient visits per week, average daily number of patients per week, and frequency of drinking per week in the previous month.

## Discussion

The associations between depressive symptoms and work environment/lifestyle among <40-year-old male orthopedic physicians in Japan were analyzed. Total working hours of ≥80 h per week at the main medical institution were associated with a depressive state. In addition, one or more unreasonable patient demands/complaints in the previous 6 months, current smoking, no sweat-inducing exercise sessions of ≥30 min per week in the previous month, and an average of <6 h sleep per night in the previous month were also associated with a depressive state. These findings suggested that the number of work hours and unhealthy lifestyle habits influenced the depressive state.

Many previous studies have reported an association between long working hours and depression, which was consistent with this study which found that ≥80 working hours per week at the main medical institution was associated with a depressive state. Proper work management and supervision are important for maintaining physicians’ mental and physical health because long working hours are an independent risk factor for depression ^(13)^. Hino et al. ^(14)^ reported that the odds ratio for the prevalence of psychological distress was significantly higher in those who worked overtime for a long time compared with those that did not work overtime. In addition, a study that investigated the relationship between weekly working hours and depressive symptoms among residents ^(15)^ found an association between ≥80 working hours per week and depressive symptoms. Moreover, working ≥80 h per week was also found as a risk factor for depression. In addition, when compared by working hours, the odds ratio for working ≥100 h was higher than that for 80-99 h (2.06 vs. 3.89), and the group working ≥100 h per week was at higher risk. Shortened working hours have also been reported to lower the level of depressive symptoms ^(16)^, suggesting that working ≥80 h a week should be avoided.

Some studies involving physicians have reported an association between the number of night duties and depressive symptoms. The burden of night duty for a doctor depends on various factors, such as busyness and whether sleep time can be secured. In particular, the burden of orthopedic night duty varies depending on the hospital size and system (e.g., whether the night duty is an orthopedic-only shift, is incorporated into a surgical night duty, or is for tertiary emergencies). Sleep deprivation due to night duties has been reported to lead to errors and poor performance ^(17)^. A study involving spine surgeons suggested that surgery immediately after night duty carried a risk for dural puncture ^(18)^. Thus, acknowledging that night duty is a risk factor for depression is necessary because reports exist that similar errors are associated with the risk for depressive symptoms ^(17)^. However, this study found no statistically significant association between the number of night duties in the previous month at the main medical institution and depressive state. Reports exist that people with illnesses are exempt from night duty, which could explain the lack of strong evidence for the association between night duty and depression ^(19)^. Further studies are needed regarding the association between night duty and mental health among physicians.

Previous studies have also reported that work that requires frequently being on call causes stress and is associated with depressive symptoms. In this study, being on call ≥10 times in the previous month at the main medical institution increased the risk of being in a depressive state. However, the result was not statistically significant. A previous study ^(20)^ found an association between depressive symptoms and being on call more than five times per month. As with night duty, the burden of being on call depends on the hospital’s size and system. If an orthopedic doctor is not stationed as a doctor on night duty, the on-call physician is often called to determine the need for emergency surgery, respond to fracture patients, and confirm instructions from the orthopedic ward. Therefore, measuring the burden on doctors simply by the number of on-call duties is not possible. Heponiemi et al. ^(21)^ reported that on-call work in addition to stressful work exacerbated depressive symptoms. On-call work affects sleep as well as depressive symptoms, which tends to adversely affect work performance and interpersonal relationships ^(22)^. Therefore, considering the on-call system and frequency of being on call when examining the burden on doctors is necessary.

Medical malpractice and complaints from patients have been reported to be associated with depression in doctors. In this study, the number of unreasonable patient demands or complaints in the previous 6 months was associated with depressive symptoms. Jain et al. ^(23)^ reported that complaints can have various effects on doctors including shock, depression, suicide, and doubts about one’s clinical performance. A UK study ^(24)^ reported that 16.9% of doctors who received complaints in the previous 6 months had depression and were at higher risk for depression compared with doctors without complaints. Wada et al. ^(1)^ also found that receiving one or more unjustified complaints over the previous 6 months was associated with depressive symptoms. In the current study, 47.2% of physicians had experienced unreasonable patient demands or complaints in the previous 6 months, and this was associated with depressive symptoms. A study involving orthopedic trainees ^(10)^ stated that preventing depression by establishing a program that responds to complaints and that these complaints should be handled by the organization is necessary.

Many studies have found a relationship between smoking and mental illness ^(25), (26)^. Similarly, this study also found associations between smoking habits and depressive symptoms. Several hypotheses were noted for this relationship and bidirectionality has been noted, such as people smoking to relieve symptoms (e.g., anxiety) ^(25)^ and smoking increasing sensitivity to environmental stressors, which can lead to depression and anxiety through the impact on neural circuits ^(26)^. A meta-analysis conducted by Taylor et al. ^(27)^ found that quitting smoking was associated with improved depression and anxiety symptoms compared with continuing smoking, and quitting smoking was as effective as or better than antidepressant treatment for people with mental illnesses.

Studies suggest that physical activity improves sleep quality, and both sleep quality and physical activity are associated with the risk of depression ^(28)^. In this study, a stronger association with depression symptoms was found in the group that had no sweat-inducing exercise of at least 30 min per week compared with the group that exercised one to two times. Zan et al. ^(29)^ reported that those who did not exercise more than once a week had a 1.6-fold higher risk of developing depression than those who exercised. In addition, Wada et al. ^(30)^ noted that those with physical activity of less than once a week had a 1.18 times higher risk for developing depressive symptoms than those who exercised. A possible reason for this finding is that physical activity causes physiological changes such as improvement of sleep efficiency and shortening of sleep onset latency, which affects sleep quality, and physical activity improves depressive symptoms and increases self-esteem ^(31)^.

An association between sleep deprivation and depressive symptoms has previously been reported ^(30)^. In this study, an average sleep of <6 h per night was also associated with depressive symptoms. The risk of developing depression may be significantly higher among those with short sleep time, especially if they have a persistent sleep disorder ^(32)^. Depression is expected to cause sleep disorders. However, sleep disorders are also considered a risk factor for depression ^(33)^, and sleep disorders and depression are thought to interact. A report involving surgeons ^(34)^ found no significant difference in complications in daytime surgery performed immediately after night duty but found a significant difference in complications in night-time surgery performed when surgeons had <6 h of sleep.

### Limitations

Drawing conclusions about causal factors was not possible because this study used a cross-sectional design. People with depression have reduced ability to concentrate thus, affecting their efficiency at work. This may result in longer working hours. Similarly, depressed people are likely to have deprivation of sleep. Therefore, whether factors such as long working hours, fewer sleeping hours, and smoking habits caused depressive symptoms or if these were the consequence of being in a depressive state is not clear. In addition, the actual burden on physicians should include not only the working hours at the main medical institution but also the working hours at other institutions. However, the actual burden may be even greater because this survey does not investigate working hours at other institutions. Other limitations of this study include the possibility that the nonresponse group had worse depressive symptoms and a relatively low response rate (response rate, 40%). When conducting similar surveys, implementing strategies to increase the response rate will be necessary. However, mentioning only this questionnaire is difficult because many factors for depressive symptoms were observed.

### Conclusion

An analysis was conducted on <40-year-old male orthopedic physicians in Japan. This study found that work factors such as total working hours of ≥80 h per week at the main medical institution are associated with a depressive state. In addition, associations between depressive state and unhealthy lifestyle factors, such as smoking habits, lack of exercise, and <6 h of sleep per night, were found. This is the first survey conducted on the working conditions of <40-year-old orthopedic physicians in Japan. However, further studies are necessary to improve the work environment and lifestyle of young physicians.

## Article Information

### Conflicts of Interest

None

### Sources of Funding

This work was supported by the Japan Orthopedic Association Gender Equality and Work System Reform Committee.

### Acknowledgement

The authors would like to express deep gratitude to the doctors who cooperated with this survey (all regular Japan Orthopedic Association members) and the doctors of the Japan Orthopedic Association Gender Equality and Work System Reform Committee.

Edanz Group is thanked for editing a draft of this manuscript.

The authors are grateful to Ms. Bibha Dhungel from St. Luke’s International University for her expertise and assistance in revising the manuscript.

### Author Contributions

A.T. and K.W. participated in the design of the study, acquisition of data, conducted the initial analyses, drafted the initial manuscript, and approved the final manuscript as submitted.

N.T., K.H., Y.M., A.O., E.H., and J.I. conceived the ideas, collected the data, revised, and approved the final manuscript as submitted.

### Approval by Institutional Review Board (IRB)

Ethical considerations related to the survey were reviewed and approved by the Ethics Review Committee established by the Japanese Orthopaedic Association and the International University of Health and Welfare Ethics Committee (Approval Number 19-Im-012).
